# SNP Discovery in European Anchovy (*Engraulis encrasicolus*, L) by High-Throughput Transcriptome and Genome Sequencing

**DOI:** 10.1371/journal.pone.0070051

**Published:** 2013-08-01

**Authors:** Iratxe Montes, Darrell Conklin, Aitor Albaina, Simon Creer, Gary R. Carvalho, María Santos, Andone Estonba

**Affiliations:** 1 Department of Genetics, Physical Anthropology and Animal Physiology, University of the Basque Country UPV/EHU, Leioa, Spain; 2 Department of Computer Science and Artificial Intelligence, University of the Basque Country UPV/EHU, San Sebastian, Spain; 3 IKERBASQUE, Basque Foundation for Science, Bilbao, Spain; 4 Molecular Ecology and Fisheries Genetics Laboratory, School of Biological Sciences, Environment Centre Wales, Bangor University, Gwynedd, United Kingdom; 5 AZTI Tecnalia, Marine Research Division, Pasaia, Spain; Harvard Medical School, United States of America

## Abstract

Increased throughput in sequencing technologies has facilitated the acquisition of detailed genomic information in non-model species. The focus of this research was to discover and validate SNPs derived from the European anchovy (*Engraulis encrasicolus*) transcriptome, a species with no available reference genome, using next generation sequencing technologies. A cDNA library was constructed from four tissues of ten fish individuals corresponding to three populations of *E. encrasicolus,* and Roche 454 GS FLX Titanium sequencing yielded 19,367 contigs. Additionally, the European anchovy genome was sequenced for the same ten individuals using an Illumina HiSeq2000. Using a computational pipeline for combining transcriptome and genome information, a total of 18,994 SNPs met the necessary minor allele frequency and depth filters. A series of further stringent filters were applied to identify those SNPs likely to succeed in genotyping assays, and for filtering of those in potential duplicated genome regions. A novel method for detecting potential intron-exon boundaries in areas of putative SNPs has also been applied *in silico* to improve genotyping success. In all, 2,317 filtered putative transcriptome SNPs suitable for genotyping primer design were identified. From those, a subset of 530 were selected, with the genotyping results showing the highest reported conversion and validation rates (91.3% and 83.2%, respectively) reported to date for a non-model species. This study represents a promising strategy to discover genotypable SNPs in the exome of non-model organisms. The genomic resource generated for *E. encrasicolus*, both in terms of sequences and novel markers, will be informative for research into this species with applications including traceability studies, population genetic analyses and aquaculture.

## Introduction

European anchovy (*Engraulis encrasicolus,* L.1758) is a small pelagic teleost with major economic and cultural importance. It has been the focus of numerous ecological and genetic studies and major research efforts have been conducted to understand population dynamics from an ecological point of view. Genetic studies have been focused on population genetic structure [1], [2], phylogeography [3], [4], species traceability [5], [6] and marker discovery [7], [8]. However, despite the economic and ecological importance of this species, there is a deficiency in available genomic information for *E. encrasicolus,* and this issue has impeded progress in molecular marker development.

Genetic markers are important for many applications [9]. More specifically, SNP markers are very informative for population assignment (facilitating the identification of migrants and estimation of current rates of dispersal), for estimates of effective population size (Ne, an important concept in the management of threatened species), and for detecting significant reductions in population size or population bottlenecks (informative in populations which have suffered a collapse) [10]. The unraveling of these questions in the European anchovy is essential, especially for the Bay of Biscay population, which suffered a collapse during last decade. The potential loss of genetic variability, with consequent reduced adaptability, population persistence, and productivity is unknown.

The advance in the genetics fields of the European anchovy had reached a plateau until the development of the next generation sequencing (NGS) technologies, which has now made possible the implementation of SNPs as standard genetic markers in non-model species [11]. In a recent non-NGS study, 62 SNPs (including 47 nuclear and 15 mitochondrial) were validated for European anchovy using random cloning and comparative Sanger sequencing [3]. This study provided insights into European anchovy population structure [2], describing ten homogeneous population groups. In some cases, these populations differed from the stocks currently defined for management in the species. For marine exploited species, a better understanding of the population structure is relevant since the chief value of genetic data to management is the identification of demographically independent populations with different patterns of recruitment, mortality and productivity [12]. This traditional method for SNP discovery (cloning and comparative Sanger sequencing) has been used for decades, but it can be expensive and time-consuming. The number of validated (meaning reliably scored and polymorphic) SNPs is still scarce and further SNP discovery would reinforce their application in prospective studies in the fields of population genetics, traceability, aquaculture and conservation.

In recent years, next generation sequencing (NGS) technologies have emerged as a cost-effective way to very rapidly generate a large amount of valuable genomic information [13] and for discovering SNPs in non-model organisms [9], increasing throughput and reducing the cost and time involved in SNP development. This is particularly crucial for non-model species with limited or no available genomic resources. For SNP discovery in non-model organisms, a “genome reduction” step may be applied in order to increase genome coverage and reach the deep assemblies of redundant reads required for SNP detection [14]; a practical and popular approach for SNP discovery in non-model species is based on reduction to the transcriptome. Recently, several studies have successfully used this approach for marker discovery in fish species relevant to fishery and aquaculture: catfish [15], lake sturgeon [16], rainbow trout [17], [18], lake whitefish [19], Atlantic cod [20], salmonids [21], [22], hake [23], turbot [24], Atlantic herring [25] and Pacific herring [26]. Indeed, transcriptome sequencing has the advantage of directly identifying expressed genes, which are often the main research focus for population genetics and aquaculture [10]. The use of transcriptome sequencing to identify SNPs presents issues all of which need to be considered in a SNP selection process. For the proper identification and validation of transcriptome derived SNPs it is necessary to filter *in silico* those markers potentially close to intron-exon boundaries (IEBs), as the proximity of an intron is known to be the main cause of genotyping assay failure due to the inability to handle large PCR amplicons [15]. Therefore, genotyping primers and probes must be designed within a single exon, completely avoiding introns. Many studies of transcriptome derived SNPs discovery in non-model fishes either do not consider the IEB problem [15], [19], [21], [22], [26] or use the standard approach to BLAST the transcriptome contigs against a closely related annotated genome [23], [25] to infer IEB positions. However, for most non-model fish species, the closest related annotated genome with IEB information may be phylogenetically divergent, and therefore most of the contigs using this standard approach end up not having a significant BLAST match. The deficient prediction of IEB ultimately leads to the low SNP validation rate, reported in previous SNP discovery studies for non-model fish species. Finally, duplicated regions must also be filtered because they usually are assembled into same contig due to their high levels of sequence similarity. This might lead to paralogous sequence variants (PSVs: single nucleotide differences between duplicated *loci* in the genome but invariant at the population or species level [27]) or multisite sequence variants (MSVs: single nucleotide variants with complex characteristics due to polymorphisms within duplicated regions [27]), both of which remain indistinguishable from SNPs in the discovery process.

In the current study, we characterized the transcriptome of *E. encrasicolus* and discovered SNP markers based on the combination of transcriptome and genome information. Importantly, next generation sequences from the European anchovy genome were used to supplement the transcriptome, in a novel strategy designed to avoid IEBs as well as potential repeated regions during SNP genotyping primer design and SNP validation. This sequencing and computational pipeline, which does not require a prior application of genome assembly, has resulted in the highest conversion and validation rates reported to date in a non-model species. The overall objectives of this study are to improve current SNP discovery procedures from transcriptome sequences through a new and accurate IEB prediction pipeline which could be reproducible for another non-model species, and to validate new SNP markers to be applicable in prospective genetic studies in European anchovy.

## Materials and Methods

**Figure 1:**
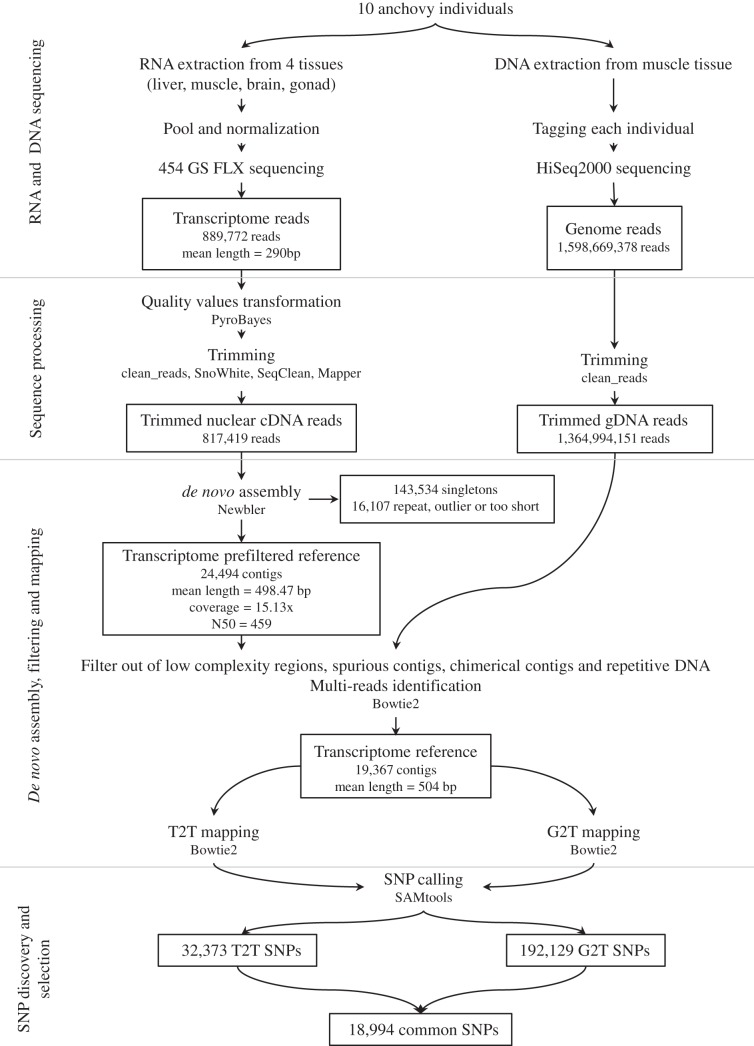
Flowchart for *Engraulis encrasicolus* SNP discovery.

The flowchart in Figure 1 summarizes the methodological process described in this section by showing the main steps of RNA and DNA sequencing, sequence processing, de novo assembly, filtering and mapping, SNP discovery and selection.

### Sample collection

**Figure 2:**
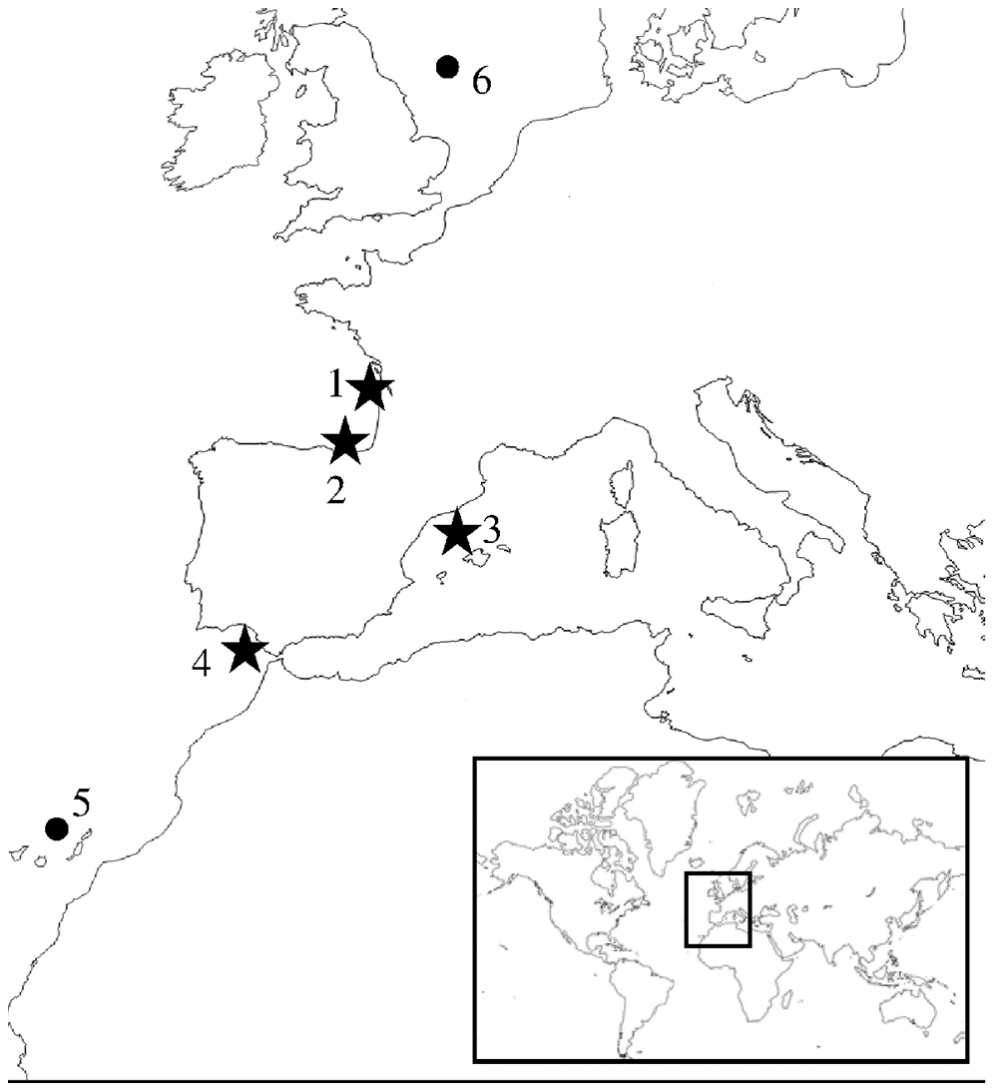
Map with sampling locations. Stars indicate sample locations used for 454 GS FLX and HiSeq2000 sequencing: 1 (BIS2; N = 2) and 2 (BIS1; N = 3) represent sampling points from Bay of Biscay population, 3 (TAR; N = 2) is the sampling point from Mediterranean population and 4 (CAD; N = 3) is the sample from the Atlantic population. Every sampling point (stars and black dots) was used for validation including N = 30 individuals. Apart from locations 1–4, two additional populations were included in this step: 5 (CAN) is the sampling location for Canary Islands population and 6 (NOR) is the sample representing North Sea population.

Ten individuals from three genetically divergent populations [2] were collected (Bay of Biscay (BIS1 and BIS2), Mediterranean Sea (MED) and Atlantic (CAD), Figure 2). Brain, gonad, muscle and liver tissues from each fish were immediately conserved in RNAlater after collection and stored at −20°C until further processing.

All surveys followed local regulations and guidelines for such research. For Spanish territories, no specific permission is needed for sampling aquatic fauna for scientific objectives, and the European anchovy is not considered a threatened species according to the International Union for Conservation of Nature and Natural Resources (IUCN Red List of Threatened Species, www.iucnredlist.org). For surveying in French territories permission was received from the French Ministère des Affaires Etrangères et Européennes (document no. 1233/DGM/ATT/ENT). Anchovies were collected following fishing without unnecessary suffering of the animals and following usual procedures: samples were obtained as part of faunal surveys with trawl nets; immediately after collection, anchovies are sorted from the bulk of the catch and a sample of 2 kg was selected at random, for which extracted tissues of 30 individuals were stored in ethanol or at −20°C. No experimentation with live animals was performed. No other ethical issues applied to the present research project.

### RNA and DNA sequencing

For transcriptome sequencing, total RNA from the ten sampled individuals and four tissues was extracted using Trizol Reagent (Invitrogen) and quantified with Agilent 2100 Bioanalyzer combined with Agilent RNA 6000 Nano chips (Agilent Technologies, Inc.) at the Gene Expression Unit (SGIker) at the University of the Basque Country (UPV/EHU). Isolated RNA (four tissues for ten individuals) was combined in equimolar quantities into a single pool in an attempt to maximize the diversity of transcriptional units sampled and RNA was normalized by Evrogen (Russia) to prevent over-representation of the most common transcripts, using the DSN normalization method [28]. The normalized RNA pool was used for double-stranded (ds) complementary DNA (cDNA) synthesis following the Evrogen CS010–1C protocol using SMART technology [29], and was precipitated as recommended by Evrogen. cDNA libraries for 454 sequencing were prepared from the normalized and digested cDNA pool according to Roche's protocol (cDNA Rapid Library preparation protocol). Finally, 454 sequencing was performed at the Centre for Genomic Research at the University of Liverpool (United Kingdom) on one half of a PicoTiterPlate^TM^ using the 454 GS FLX Titanium System (454 Life Sciences, Branford, CT, USA). All 454 sequence data have been submitted to the NCBI Sequence Read Archive (SRA) under the BioProject accession number PRJNA193183.

For the genome sequencing, total DNA from muscle tissue of ten individuals (the same individuals used for transcriptome sequencing) was isolated using NucleoSpin® 96 Tissue Kit (Macherey-Nagel) according to the manufacturer's instructions and DNA quantity and purity were measured with a Nanodrop ND-1000. The Illumina TruSeq® DNA sample preparation kit was used to generate a barcoded genomic library for each individual. The ten barcoded genomic libraries were pooled and sequenced with a 2×100bp paired-end module on 4 lanes of a HiSeq2000 (Illumina). Standard post-processing was applied (adaptor clipping and quality checking) and sequences were de-multiplexed based on the specific barcoding tags used for each individual. The genome sequencing and post-processing was carried out at the Laboratory of Biodiversity and Evolutionary Genomics at the Katholieke Universiteit Leuven (Belgium). All HiSeq2000 sequence data have been submitted to the NCBI Sequence Read Archive (SRA) under the BioProject accession number PRJNA202430.

### Sequence processing

To improve the 454 native base-calling error rate, PyroBayes [30] was used to transform the native 454 quality values into the standard Phred64 quality scores [31]. Following this, transcriptome raw reads were trimmed using clean_reads [32] and SnoWhite with the TagDust option [33]. In this trimming process SMART adaptors, PCR primer sequences, and poly(A/T) tails were removed, and a quality and length-based trimming was done according to custom parameters. Trimmed cDNA reads were aligned against the *E. encrasicolus* mitochondrial genome (NCBI Accession Number: AP009137) to identify and isolate all mitochondrial transcripts using GS Reference Mapper v2.6 (‘Mapper’, 454 Life Sciences) with custom parameters. Additionally, a local BLASTn search on the trimmed dataset was performed to identify ribosomal transcripts (homologous to teleostei ribosomal gene nucleotide sequences) and the SeqClean tool was used to screen out UniVec database contaminating sequences.

The European anchovy genome raw reads were trimmed using clean_reads [32] for quality and length-based trimming according to custom decided parameters, as well as for screening out UniVec database contaminating sequences. Additionally, quality was visually checked before and after the trimming process with the FastQC tool.

### *De novo* assembly, filtering and mapping

Transcriptome trimmed sequences were assembled using GS *de novo* Assembler v2.6 (454 Life Sciences) with the cDNA assembly option, by setting a *minimum overlap length* of 50 and a *minimum overlap similarity* of 95%. Reads were re-trimmed with the GS *de novo* Assembler trimming tool, including in the GS *de novo* Assembler *exclude filter file* the previously created file with names from cDNA reads corresponding to mitochondrial, ribosomal or contaminant sequences. The assembly quality was verified by visual examination of a random subset of contigs with the Tablet assembly and alignment visualization tool [34].

A transcriptome reference assembly was created by filtering transcriptome contigs for (1) homologous genes, (2) low-complexity regions and (3) duplicated regions. For the removal of homologous genes (two or more potentially homologous genes which are incorrectly assembled into one contig) and low-complexity regions, gDNA reads were aligned to the transcriptome reference using Bowtie2 [35] and contigs with a disproportionately large number of aligned gDNA reads were removed. For the avoidance of duplicated regions, an additional filter based on the identification of multimap reads was applied. Multimap reads are defined as those gDNA reads that align to multiple positions in the transcriptome reference, and as a consequence can suggest ambiguous or repetitive regions [36]. For detecting multimap reads, an alignment was performed using Bowtie2 (setting k = 2, local alignment mode). Then, contigs containing one or more multimap reads (detected by inspecting the bitwise FLAG string of each read alignment within the SAM file produced by Bowtie2) were defined as potentially duplicated regions and removed for posterior analyses.

Finally, Bowtie2 [35] was used to create two mappings to the transcriptome reference for SNP discovery purposes. In the first mapping, called G2T (genome to transcriptome), gDNA reads were aligned to the transcriptome reference, and in the second mapping, called T2T (transcriptome to transcriptome), cDNA trimmed reads were aligned to the filtered transcriptome reference. Both mappings (G2T and T2T) are referenced during the SNP discovery and selection process.

### SNP discovery and selection

With the aim of avoiding false SNPs (monomorphic *loci*) due to sequencing errors, SNP discovery was performed using both T2T and G2T alignments. For SNP discovery and selection we developed a method for automatically extracting SNPs amenable to genotyping from the SAM output files of the T2T and G2T alignments, and the VCF (variant call format) output of the SAMtools package [37].

For SNP discovery, the T2T and G2T alignments were processed with SAMtools bcftools and duplicated reads (arising from errors in the PCR step prior to sequencing) from each dataset (cDNA and gDNA trimmed reads) were removed with rmdup option. Putative SNP discovery was accomplished by filtering all T2T and G2T observed variants in order to reveal only biallelic SNPs (no indels or complex SNPs). To avoid false positives due to sequencing errors (which may therefore be monomorphic *loci*), only T2T variants with a minimum variant count of 2 high quality (HQ) bases and a minimum site depth of 8 (HQ bases) were called as putative T2T SNPs (no depth filter was applied for a variant count of 3 HQ bases or more). In parallel, from the G2T SNPs only variants with a minimum variant count of 2 HQ bases and a minimum site depth of 20 (HQ bases) were called, but no limitation of depth was required for a variant count of more than 2 HQ bases. Finally, a maximum site depth threshold of 200 for each SNP was applied. This final step was performed also with the aim of avoiding duplicated regions due to homologous genes (PSVs or MSVs).

**Figure 3:**
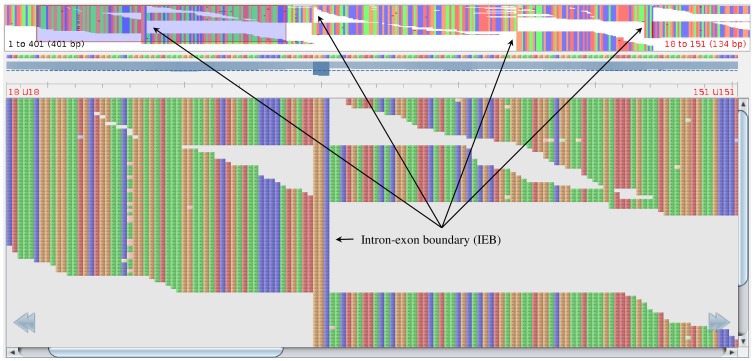
Output from the Tablet alignment visualizer [34] showing a G2T alignment for which 4 IEBs (arrows) have been detected (upper part of the display). The bottom part of the display focuses on the magnified area around the first IEB alignment pattern. See Materials and Methods for further explanation.

For each filter (T2T and G2T) all SNPs meeting the respective requirements described above were marked and the T2T and then the G2T SNPs sets were joined, resulting in only those SNPs discovered from both approaches. After the SNP discovery step, SNPs were *in silico* assessed to select several SNP markers for validation with the TaqMan® OpenArray^TM^ platform (Life Technologies). The selection criteria for SNPs were based on an analysis of putative intron-exon boundaries (IEBs) within each contig and on the compatibility of flanking sequence with the Taqman® method. Initial efforts of IEB finding through a BLAST search of European anchovy contigs against other teleost genomes showed that most contigs had no significant matches to other teleost genomes. Therefore, a novel approach to IEB detection [38] was designed, which can be applied when gDNA reads are available. The basis of this algorithm is the observation that the alignment of genome reads to a transcriptome contig produces distinctive patterns at the areas of IEBs, which emerge as *change points*: locations where a number reads either start or terminate their local alignment at an internal position (Figure 3). Taking advantage of this property, the algorithm processes the G2T SAM files (using the Perl Bio-Samtools library v1.36), computing a p-value for every change point within a contig. Low p-values indicate an unexpectedly large number of reads supporting the change point, and are suggestive of IEB (for example, for the contig shown in Figure 3 all indicated locations have a computed p-value less than a calibrated discriminative threshold, with no other location below this threshold), and every contig was divided into several sequences (putative exons) using each predicted IEB as a breakpoint. Second, for designing 530 TaqMan® OpenArray^TM^ SNPs genotyping assays, one sequence was built for each detected SNP. In that sequence, every putative SNP but the target was masked as *N*s, including those called in only one approach), which were also masked as *N*s in every sequence. We ensured that target sites were not in regions of similarity with any other contig in the European anchovy transcriptome, as identified by a BLASTn search (E-value <10^−25^). Based on the genotyping technology requirements, we rejected any sequence with less than 30 available bases upstream or downstream of the target site (due to the start/end of the contig or due to a predicted IEB), to comply with the minimum requirement for primer design, and any target site without complete alignment conservation (no variation or sequencing *N*s) within +/−5 bases.

For TaqMan^®^ OpenArray^TM^ SNP genotyping assay construction, SNPs were divided by two selection criteria. A first SNP subset was selected based on homology to zebrafish (*Danio rerio*) exome. For this, conservation of gene structure between European anchovy and zebrafish was assumed, and each contig was assessed using a database of coding sequences for all transcripts from the Ensembl zebrafish genome (Zv9). Anchovy filtered transcriptome contigs were aligned at the protein level using BLASTx (e-value <10^−10^) to identify a possible unique orthologous zebrafish gene. SNPs were annotated in 4 categories: *no homology* (no homology found to zebrafish), *cSNP* (contig with homology and SNP in a coding region), *ncSNP* (contig with homology and SNP outside of coding region) and *tSNP* (ambigious cases where a contig has a homology to a coding region and another homology outside a coding region). All *cSNPs* were selected and further SNPs were randomly selected from the *tSNP*s list in order to have potentially coding SNPs, interesting for adaptative and evolutionary studies. For the second SNP subset, a preliminary Fst value was calculated for suggesting how well the SNPs may discriminate populations. In this case, individual genotypes for each called SNP were determined by inspecting the VCF output of bcftools. This genotype information was used to calculate a preliminary Fst value [38], [39] for each marker in the 10 individuals from three populations. Markers were sorted from high to low Fst and those SNPs of higher Fst values were selected.

### SNP genotyping and validation

A total of 180 samples of *E. encrasicolus* from five different populations (Bay of Biscay (BIS1 and BIS2), Mediterranean Sea (MED), Atlantic (CAD), Canary Islands (CAN) and North Sea (NSE); (Figure 2) according to [2] were used for genotyping and validating the 530 selected SNPs. From each population 30 individuals were included in the study, although 60 individuals were genotyped from Bay of Biscay (30 individuals from 2 sampling locations, BIS1 and BIS2; Figure 2). DNA extractions were performed from muscle tissue using NucleoSpin® 96 Tissue Kit (Macherey-Nagel) according to manufacturer instructions, and DNA quantity and purity were measured using Nanodrop ND-8000.

A submission file with the sequences specifying the target SNPs was created and sent to the Applied Biosystems Assay Service for primer and probe design. Genomic DNA (66 ng per sample) was used as template at the required DNA starting concentration (22 ng/µl). Subsequent reactions for the amplification and detection of the SNPs were carried out following *TaqMan® OpenArray™ Genotyping System User Guide* at the Sequencing and Genotyping Service (SGIker) of the University of the Basque Country (UPV/EHU). Scoring of individual genotypes was performed using TaqMan*®* Genotyper software v2.1 (Life Technologies). After default clustering was performed, data was viewed in the scatter plot and genotype calls were reviewed and manually adjusted for producing the final cluster assignments. Based on these assignments, SNPs were classified as *no signal* (no amplification), *disperse* (less than 80% of individuals assigned to a cluster), *monomorphic* (minor allele frequency, MAF <0.01), *PSV/MSV* (≥99% of all individuals heterozygous), and *polymorphic* (MAF ≥0.01).

Once *no signal* and *disperse* SNPs were removed, the remaining SNPs were used for the calculation of the SNP conversion rate, while only *polymorphic* SNPs were used for the calculation of SNP validation rate and for further analysis of descriptive statistics.

For each polymorphic SNP, the genotyping percentage was calculated using Genepop v4.0 [40]. For each polymorphic locus and population, Fisher's exact test was used to test deviations from Hardy-Weinberg equilibrium (HWE) across samples, as implemented in Genepop v4.0 (p-value <0.001). Moreover, in order to identify only independent markers, linkage disequilibrium (LD) was tested with *genetics* package from R [41]. Then, for each independent marker, the expected heterozygosity (H_e_), observed heterozygosity (H_o_), and minor allele frequency (MAF) were calculated using GeneClass2 [42]. The BayeScan 2.1 software [43] was used to identify candidate *loci* under natural selection. In this test, because multiple comparisons were involved, critical values for the test were adjusted with false discovery rate (FDR) procedure (q-value <0.1) [44].

### Microsatellite loci discovery

Transcriptome contigs were independently searched for microsatellite repeats and primers using the software QDD2 [45]. Additionally, repeats and their flanking sequences were BLASTed against the non-redundant section of NCBI (nt) for identifying the taxonomic lineage of the organism of the best hit. The microsatellite set was filtered for those meeting the following criteria: (1) at least five uninterrupted repetitions for di-, tri-, tetra-, penta-, and hexa-nucleotides, (2) a pure motif and (3) a primer design “A”, which means that neither homopolymers nor nanosatellites were allowed in the primer sequence, or in the target microsatellite flanking region.

### Gene annotation

We used BLASTx (E-value <10^−6^) to align the contigs to the manually curated protein database Uniprot/Swissprot [46] using Blast2GO tool [47] against the zebrafish proteome. Blast2GO is an automated tool for the assignment of gene ontology terms to BLAST hits, designed for use with novel sequence data [47]. Assignment of gene ontology terms to contigs with significant BLASTx match was also performed using Blast2GO.

## Results

### RNA and DNA sequencing

Anchovy transcriptome sequencing yielded 889,772 reads with a length average of 293 bp comprising a total of 244 Mbp. Anchovy genome sequencing produced 1,598,669,378 paired-end sequences of 100 bp for each read.

### Sequence processing

Raw transcriptome reads were trimmed (see Materials and Methods section) resulting in 821,107 reads with a length average of 304 bp comprising 225 Mbp. These trimmed sequences were considered as high-quality (HQ) sequences (92.3% of raw reads). Only nucleotides with Phred “high quality bases” were considered (HQ Phred score >20) [48]. A total of 1,446 mitochondrial transcriptome sequences were identified, matching 93% of the mitochondrial genome reference with a coverage (mean depth per reference base) of 24.51. Additionally, 1,701 reads were found to have a BLASTn hit with teleostei organism ribosomal RNA sequences. Finally, 1,000 reads were removed due to a BLASTn hit to the UniVec database. The exclusion of all these sequences resulted in removing a total of 3,688 reads from the trimmed dataset. Therefore, a completely trimmed dataset of 817,419 (91.9% of raw reads) anchovy nuclear transcriptome reads were obtained for anchovy nuclear transcriptome *de novo* assembly.

Genome sequencing contaminant removal, and length and quality trimming, yielded 1,364,994,151 HQ paired-end sequences (85.4% of initial reads), with read lengths ranging from 61 to 100 bp. The trimming results for each individual are detailed in Table 1. In terms of sequencing success, individual GIR-4 had the highest percentage of valid sequences (93.7%); individual CAD-1 (182,750,832 trimmed sequences) had the highest number of valid sequences, and was also the one with the highest number of sequenced raw reads (211,055,936 sequences). Positions 70 to 100 of genome reads had quality values as low as 0 that were completely removed after the trimming process (Figure S1). After sequence cleaning, read quality values were also lower at the beginning and the end of the sequences, but quality average always stayed between 30 and 40, defined as *high-quality region*. FastQC tools analyses showed that the trimming process substantially improved the genome sequence dataset, especially in terms of sequence quality, which is essential for the success of the SNP calling process.

**Table 1:** Sequenced individual, number of sequences obtained from HiSeq2000 sequencing (Raw sequences), number of trimmed sequences, and percentage of valid (or trimmed) sequences.

Individual	Raw sequences	Valid sequences	Valid sequences (%)
BIS2–4	124,890,134	116,962,879	93.65%
BIS2–5	136,203,704	120,970,864	88.82%
BIS1–3	203,056,696	180,588,178	88.93%
BIS1–4	199,138,100	153,625,124	77.15%
BIS1–5	125,842,196	111,469,177	88.58%
TAR-4	166,733,374	144,736,461	86.81%
TAR-6	160,620,594	142,320,609	88.61%
CAD-1	211,055,936	182,750,832	86.59%
CAD-3	181,504,151	160,144,743	84.82%
CAD-5	151,952,366	128,884,436	69.38%
TOTAL	1,598,669,378	1,364,994,151	85.38%

### *De novo* assembly, filtering and mapping

GS *de novo* Assembler software assembled 657,778 reads (80.5% of trimmed reads) into 24,494 contigs with a N50 of 459, and an average coverage of 15.13. The assembled contigs had an average length of 498bp, comprising more than 180 Mbp. The longest contig was 3,336bp length and 10,095 contigs (41.2%) were longer than 500 bp. The N50 of these large contigs was 766. The total assembled reads length was 12,209,523bp. Reads that were not assembled constituted 143,537 singletons (17.6%), which were excluded for further analyses. The remaining 1.9% of reads was identified by GS *de novo* Assembler software as repeats, outliers, or reads too short for use in the assembly. The transcriptome reference was filtered for low-complexity regions, spurious contigs, chimeric contigs, and duplicated regions in the genome, yielding a total of 19,367 high-confident consensus sequences. From these, 10,402 contigs containing multimap reads were identified. In the T2T alignment, 593,122 reads successfully mapped, with a coverage of ∼15. For the G2T local alignment, 11,361,696 reads aligned one or more times, with a coverage of ∼78.

### SNP discovery and selection

A summary of SNP discovery and selection statistics is presented in Table 2. From 41,542 T2T biallelic variants, 32,373 remained after filtering based on the minimum variant count and site depth threshold. For G2T biallelic variants, 208,016 were filtered based on the maximum site depth threshold, resulting in 192,129 SNPs. Within the T2T and G2T SNPs sets, 18,994 were common to both. These SNPs were found within 7,426 distinct contigs. Transitions were the most common SNP type, with a ts/tv ratio of 2.31. Regarding IEB avoidance, a total of 4,031 of the 7,426 contigs with a common SNP contained one or more predicted IEB (Table 2), and a total of 14,186 IEBs were identified in these 4,031 contigs (on average 3.52 predicted IEB per contig). These predicted IEBs were annotated and further avoided.

**Table 2:** Summary statistics of SNP discovery and selection.

			T2T	G2T	
Biallelic variants			41,542	208,016	
*In silico* putative SNPs (after filters)			32,373	192,129	
Contigs with putative SNPs			13,671	17,406	
Total predicted IEB			10,688		
	contigs with one or more predicted IEB		4,031		
Common SNPs			18,994		
	contigs with a common SNP		7,426		
	transitions		13,255		
	transversions		5,739		
	SNPs suitable for TaqMan® OpenArray^TM^		2,317		
		*cSNPs*	195		
		*ncSNPs*	423		
		*tSNPs*	274		
		*no homology*	1,425		
Selected for validation			530 (100%)		
	failed				
		*disperse*	16 (3.0%)		
		*no signal*	30 (5.7%)		
	false				
		*monomorphic*	40 (7.5%)		
		*PSV/MSV*	3 (0.6%)		
	*polymorphic*		441 (83.2%)		

TaqMan® OpenArray^TM^ SNPs genotyping system requirements were passed by 2,317 SNPs which appeared within 1,617 contigs. From these, 892 contigs (55.16%) showed homology to the *D. rerio* proteome: 195 *cSNPs* (21.86%), 423 *ncSNPs* (47.42%) and 274 *tSNP*s (30.72%); for the first SNP subset every *cSNP* was selected and it was completed with markers annotated as *tSNPs*. Regarding the SNP selection criteria based on preliminary Fst, the second SNP subset consisted on those SNPs – not coincident with those selected from the zebrafish homology criteria – with the highest Fst values (from 0.31 to 0.83) were selected. The two SNPs subsets led to a total of 530 SNPs for genotyping and validation.

### SNP genotyping and validation

The final set of selected and genotyped 530 SNPs is listed in Table S1, and results are shown in Table 2. A total of 484 SNPs amplified and produced clear clusters (16 *no signal*, 30 *disperse*); which constitute a conversion rate of 91.3%. From those SNPs, 441 were polymorphic (40 *monomorphic*, 3 *PSV/MSV*) resulting in a validation rate of 83.2%.

Information for each selected SNP can be found in Table S1. Deviation from HWE for each locus and population after correction for multiple testing revealed 15 markers retaining significant deviation. Linkage disequilibrium (LD) was assessed for each pair of *loci* meeting HWE and 11 SNPs in LD formed 5 haplotypes. Therefore a total of 420 independent markers meeting HWE were found to be potentially used in prospective studies. The expected heterozygosity (H_e_) of these markers ranged from 0.012 to 0.495, while observed heterozygosity (H_o_) fluctuated between 0.007 and 0.550, and MAF values ranged from 0.003 to 0.498. The distribution of SNPs frequencies over the range of MAF categories does not suggest an elevated non-random exclusion of SNPs with low MAF, adequately showing even representation over the entire MAF range (Figure S2). Finally, 31 candidate *loci* under natural selection were identified; 30 of these markers had positive alpha values, suggesting diversifying selection [43]; and 1 SNP showed a negative value of alpha, suggesting balancing or purifying selection [43] (Table S1).

### Microsatellite loci discovery

In total, 510 microsatellite markers matching the quality criteria implemented in QDD2 and posterior filters were detected in the European anchovy filtered transcriptome (19,367 high-confidence contigs) (Table 3). The most common motif was the di-nucleotide AC, appearing in 46.9% of detected best microsatellites. The total sequence length of di-, tri-, tetra-, penta- and hexa-nucleotide repeats found in the anchovy transcriptome was 6,692bp, representing approximately 0.1% of the total assembled transcriptome contig sequences.

**Table 3:** Distribution of microsatellite repeat sizes and lengths.

repeat type	number of repeat units						maximum repeat units	total
	*5*	*6*	*7*	*8*	*9*	*10*		
dinucleotide	204	91	37	27	8	5	10	372
trinucleotide	82	22	10	2	0	0	8	116
tetranucleotide	14	3	0	0	0	0	6	17
pentanucleotide	4	0	0	0	0	0	5	4
hexanucleotide	1	0	0	0	0	0	5	1
all	305	116	47	29	8	5		510

Approximately 10% of the detected repeats matched 49 sequences isolated from 12 different species, all of them fishes (Class *Actinopterygii*) except one amphibian (*Xenopus tropicalis*), which matched only one microsatellite sequence (e-value of 10^−45^). All detected microsatellite *loci* are listed in Table S2.

### Gene annotation

The 19,367 transcriptome contigs reference were annotated using the Blast2GO tool against the zebrafish proteome. The BLASTx analysis resulted in 6,100 sequences with at least one BLASTx hit. This result indicated that 31.5% of *E. encrasicolus* transcriptome sequences could be annotated with a putative function (E-value <10^−6^). These sequences were assessed for gene ontology terms (with low MAF, adequately showing even representation over the entire MAF range (Figure S3). The vast majority of genes, within the biological process category, were included in the categories of *cellular* (18.6%), *metabolic* (15.9%) and *biological regulation* (11.1%). Most molecular functions found in this study were related to *binding* (16.7%) and *catalytic activity* (10.8%). Finally, regarding the cellular component gene ontology category, the most common components were the very general term *cell* (19.6%) and *organelle* (16.5%).

## Discussion

This study presents one of the largest combined (transcriptome and genome) sequencing projects for a non-model species and is the most extensive genomic analysis performed on the ecologically and economically important *E. encrasicolus*. Moreover, we report the highest SNP conversion and validation rates described to date for a non-model species, demonstrating a method for rapid and cost-effective SNP discovery in the exome of non-model organisms.

The success on the recovery and validation rates of the SNP markers in the present study relies on the strategies adopted to (1) avoid ascertainment bias, (2) trim and quality filter the transcripts, (3) establish criteria for accurate SNP calling and (4) accurately identify duplicated regions and intron-exon boundaries.

Regarding ascertainment bias, it has been previously shown that the deviation towards detecting only SNPs with high or intermediate allele frequencies might be a problem as it influences the precision of estimates related to demographic parameters such as migration or Ne, which could lead to mistaken assumptions about demographic history of the species [49]. In this study, *E. encrasicolus* individuals from genetically distant populations [2] were sequenced (both in the transcriptome and the genome) as it has been reported that biases related to allele frequencies could be minimized if the individuals selected to discover putative SNPs are geographically, genetically, and phenotypically diverse [50], [51]. In the present study, the obtained MAF values ranged from 0.006 to 0.498 showing an even representation over the entire allele frequency range (Figure S2), which demonstrates the high efficacy of polling genetically heterogeneous samples to avoid ascertainment bias using NGS approaches and the suitability of the markers discovered to study demographic history of populations.

It is well known that one of the major challenges for SNP discovery studies in non-model organisms is to achieve a high quality *de novo* assembly for SNP discovery. To deal with this issue, in the present study, a transcriptome rather than a genome assembly was chosen as the primary substrate for SNP discovery. The chosen strategy has the additional advantage that SNP markers derive directly from exonic regions of the genome, which are especially relevant for fisheries genetics, traceability, adaptative studies, conservation studies and aquaculture applications. For non-model species, high-throughput technologies are currently the most recommended since they generate a massive quantity of sequences and, specifically, the main advantage of the 454-FLX system is the production of longer reads than other sequencing systems, which helps the *de novo* assembly step. Additionally, performing suitable bioinformatic analyses such as base-call accuracy improvement, trimming, and the assembly itself (as well as its posterior filtering), is also important. This study has carefully taken into account all these issues. First, regarding the reduction of the error rate of base calls and improvement of accuracy in quality scores, PyroBayes algorithm produced a significant improvement in terms of trimming on quality results (data not shown). This correction may have led to a significant reduction in false SNP calls and may have facilitated the assembly [52]. Second, in connection with the trimming step, mitochondrial, ribosomal and contaminant trimming from transcriptome reads was successful since no gene from these categories was assembled, as observed in *cellular component* Gene Ontology terms from the annotation step. Indeed, the beginning and the end of genome reads had very low quality values prior to the trimming process. Such a feature is common to all high-throughput sequencing methods (see [53] for a review) and additional filtering should be performed to remove low quality, very short, or highly repetitive reads [54]. The use of FastQC trimming tools analyses proved that trimming process substantially improved the genome sequence dataset, especially in terms of sequence quality, which is essential for future SNP calling. These good results may arise from the variety of software used for trimming because, although several tools have been developed for NGS sequencing data trimming (e.g. [32], [33]), each utilizes a different algorithm and has limitations, which are reduced by employing a combination of trimming tools. Finally, with regard to the assembly step, in this study a high quality *de novo* transcriptome reference has been assembled as is confirmed by the SNP validation results. The high SNP conversion rate (484/530) is a result of the carefully quality controlled assembly, since the sequence for primer and probe design was correctly constructed in at least 91.3% of the cases. The assembly issue remains challenging mainly due to two factors: first, sequencing errors can lead to mismatching sequences between reads that came from the same location in the genome/transcriptome [55]; and second, duplicated and repetitive sequences may cause omissions, or even concatenation of reads that should not be assembled into the same contig [56]. In this study, excessively short reads and sequencing errors have both been avoided through a stringent trimming based on length and quality, as recommended by [54]. Additionally, further filtering was carried out in order to remove low-complexity regions, spurious contigs, chimeras, and duplicated genome regions from the constructed transcriptome reference dataset.

The third key factor of this study is the criteria followed for putative SNP calling. One of the most important challenges for SNP discovery is to differentiate sequencing errors from potentially real differences due to polymorphisms [52]. In the absence of a reference sequence, distinguishing true polymorphisms from sequencing errors (false positives) is difficult. Therefore, a highly conservative pipeline for the rigorous avoidance of false SNPs has been performed. For this, two different sequencing technologies (each with its inherent sequencing error type), variant site depth, and the alternative allele count have been taken into account. The use of two technologies has allowed dealing with the two main problems of SNP calling from NGS technologies: (1) obtaining a sufficient read coverage and (2) the avoidance of sequencing errors. In this study, 454 platform has been used for generating a good reference, while Illumina sequencing has compensated for the coverage issue. Second, in terms of sequencing errors, 454 technology has compensated Illumina sequencing errors in order to avoid false SNP discovery. In this study *monomorphic* markers represented just 7.5% of the putative SNPs (40/530). Compared to other *de novo* transcriptome sequencing and SNP discovery studies (see Table 4 for examples in fish species), this study has the lowest false SNPs discovery rate, very similar to those reported for species with a close reference genome and it has achieved the highest validation rate (83.2%). The combination of both technologies, joined with the stringent filters applied in each of the two parallel SNP discovery strategies (G2T from Illumina reads, and T2T from 454-FLX reads) is an effective SNP discovery procedure.

**Table 4:** Approaches to transcriptome SNP discovery and validation in fish species.

Organism and study	Sequences	Putative SNPs	Conversion rate	False SNPs rate	Validation rate	IEB method	Comments
*No reference genome*							
Catfish [15]	Sanger-EST	384	69.3%	28.6%	40.6%	none	no NGS
Lake whitefish [19]		31					
Salmon [21]	454	202	40.6%	22.3%	18.3%	none	
Sockeye salmon [22]	SOLiD	96	53.1%	41.7%	11.5%	none	RRL*1
Hake [23]	454	944	43.3%	15.9%	27.4%	homology	
	GAII*2	684	43.3%	14.0%	29.2%		
Atlantic herring [25]	454	1,536	50.7%	13.1%	37.6%	homology	
Pacific herring [26]	454	96	47.9%	33.3%	14.6%	none	
This study	454 and HiSeq2000	530	91.3%	8.1%	83.2%	read mapping	
*Draft reference genome*							
Atlantic cod [59]	Sanger-EST	594	69.0%	15.5%	53.5%	none	no NGS
Atlantic cod [20]	GAII	3,072	74.6%	19.8%	54.8%	none	

*1 RRL: Reduced Representation Libraries (method for the selection of a subset of the genome for assembly).

*2 GAII: Genome Analyzer II (Illumina NGS sequencer).

The final essential component of this study is the novel bioinformatic processing for SNP discovery including PSVs/MSVs detection and a solution to the IEB problem in a non-model organism transcriptome sequencing [38]. One of the advantages of sequencing both the genome and transcriptome (which may be the target reference for SNP discovery), is the possibility of optimally exploiting genome information for detecting duplicated regions and intron-exon boundaries.

The high failure rate of SNP selection in some projects has previously been attributed to duplicated and repetitive sequences, within PSVs and/or MSVs [57]. This is because paralogs sharing high levels of sequence similarity usually will be assembled into the same contig, and SNPs become indistinguishable; but they do not provide the same information. In the present study, PSVs and MSVs were successfully avoided (0.6% of the putative SNPs). This success is a result of filtering for duplicated genome regions by three different filters applied along the study: (1) transcriptome reference filtering, (2) maximum site depth for variants discovered in the G2T approach, and (3) multimap reads identification (see Materials and Methods). Even prior to genotyping, one clue for the accuracy of our SNP discovery process arises from the observed ts/tv ratio. It is generally assumed for humans that ts/tv ratio is around 3.0 for exonic SNPs and about 2.0 elsewhere in the genome [58]. As ts/tv differs from species to species, the ts/tv ratio of 2.3 observed for exonic SNPs in this study (between 2.0 and 3.0) reflects the high precision of the SNP discovery process.

The key to achieving high SNP conversion rates from transcriptome data is the identification of IEBs and their avoidance in primer and probe sequences for posterior marker validation [15]. Until now, IEBs have been either ignored during the marker validation phase, or identified by homology of transcriptome contigs with the sequenced genome of another species through BLAST searches (see Table 4 for references). This strategy has worked well in species with a close reference genome; but it has been very weak for fish species (Table 4) because the only nine fish with complete genome coverage (*Danio rerio*, *Gadus morhua*, *Gasterosteus aculeatus*, *Latimeria chalumnae*, *Oreochromis niloticus*, *Oryzias latipes*, *Takifugu rubripes, Tetraodon nigroviridis*, and *Xiphophorus maculatus*) are too divergent from *Engraulis encrasicolus* for significant BLAST matches at the nucleotide level. For Atlantic cod transcriptome SNP discovery [20], [59], conversion and validation rates are among the highest reported in fish species, presumably due to the availability of the cod draft genome. In the present study a new IEB detection method [38] was applied and only 16 SNPs have been classified as *no signal*, which means that only the 3.0% of the putative markers have not amplified. The new approach for IEB detection described in this study provides successful detection of IEB within transcriptome assemblies while bypassing the need to construct an assembled genome, a complicated and time-consuming task.

Most studies using high-throughput sequencing technologies for discovering SNPs from non-model fish transcriptomes have obtained conversion rates between 43% and 79%, and validation rates between 12% and 55% (Table 4), which are low values considering the scale, time and money involved in such studies. Indeed, these values reduce to 43–50% and 12–38% (conversion and validation rates, respectively) excluding species with a draft genome or large genomic resources due to decades of genetic research as cod, salmon and catfish. Here, we have significantly improved conversion rates to 91.3%, accompanied by a validation rate of 83.2%. Notably, such high rates conversion and validation are among the top of the range even for studies having access to a close reference genome (77–95% and 66–95%, respectively; e.g. [60], [61]–[63]). Furthermore, our study revealed a very small number and percentage of false positives, representing only 8.11% of the 530 SNPs set (40 monomorphic and 3 PSVs or MSVs). This data reflects an improvement in SNP selection, comparable to results obtained in other non-model fish species where between 13–33% of SNPs were false positives (Table 4).

Moreover, based on the validation rates obtained on the 530 tested SNPs subset, the extrapolation of the 83.2% of validation rate obtained on the 530 tested SNPs to the whole 2,317 putative SNPs discovered, would yield around 2,000 SNP validated markers in the exonic regions of *Engraulis encrasicolus*, a non-model organism (assuming that all 83.2% of the 2,317 putative SNPs would be suitable for genotyping primer design). In all, these percentages reflect the accuracy and effectiveness of the described strategy.

## Conclusions

The SNP discovery pipeline described in this paper has identified over 19,000 putative SNPs in *E. encrasicolus*. The technique is based on a single half-plate run on a 454 GS FLX (Roche) sequencing instrument using titanium chemistry for transcriptome sequencing, and two lanes of a HiSeq2000 (Illumina) instrument for genome sequencing. This approach can be used for rapid, comparatively low-cost SNP discovery and high conversion and validation rates in any non-model organism. While the cost of the method described here is comparable to traditional alternatives for SNP discovery, the approach has the added benefit of detecting a large number of reliable SNPs in non-model organisms. As these *loci* are derived directly from transcribed sequences, gene function annotation of the discovered markers is possible and markers under selection are expected. The value of SNPs under selection for fisheries management is that these are more informative than neutral ones when aiming for population/origin assignment of individuals. Therefore these SNPs could be informative in studies on adaptation, origin assignment or aquaculture.

Regarding European anchovy, the target non-model species of this study, the 441 validated SNPs may be useful in prospective genetic studies for understanding and estimating effective population size and detecting significant reductions in population size or population bottlenecks in populations which have suffered a loss of genetic variability. Since markers linked with genes influencing fitness might generally provide a good indicator of levels of adaptive variation within populations and their potential to respond to changing environmental conditions [14], the new markers reported here could be very informative in terms of conservation studies in the European anchovy.

## Supporting Information

Figure S1 **FastQC tool generated quality plot for CAD-1 individual genome sequences before (left) and after (right) contaminants removal, and length and quality trimming**. In the plot X axis represents position in the read (bp) from 0 to 100, and Y axis represents quality values in Phred+33 scale (from 0 to 40).
(PDF)

Figure S2 **Minor allele frequencies (MAF) values obtained from 435 independent validated markers (in H-W equilibrium or not).**
(PDF)

Figure S3 **Level 2 gene ontology terms, divided in the three categories, and the percentage of** ***Engraulis encrasicolus*** **genes for each term.**
(PDF)

Table S1 **530 genotyped SNPs sequences and their descriptive statistics.** For each marker following information is provided: NCBI Submitter SNP (ss) accession numbers, reference and alternative alleles, flanking sequence, category, genotyping percentage, HWE, linkage disequilibrium (LD), expected and observed heterozygosities (H_e_ and H_o_, respectively), minor allele frequency (MAF) and natural selection state.
(XLSX)

Table S2 **Best microsatellite markers detected in the European anchovy transcriptome.**
(XLSX)

## References

[1] Zarraonaindia I, Pardo MA, Iriondo M, Manzano C, Estonba A (2009) Microsatellite variability in European anchovy (*Engraulis encrasicolus*) calls for further investigation of its genetic structure and biogeography. ICES J Mar Sci 66: 2176–2182 doi:10.1093/icesjms/fsp187

[2] Zarraonaindia I, Iriondo M, Albaina A, Pardo MA, Manzano C, et al (2012) Multiple SNP markers reveal fine-scale population and deep phylogeographic structure in European anchovy (*Engraulis encrasicolus* L.). PLoS One 7: e42201 doi:10.1371/journal.pone.0042201 22860082 10.1371/journal.pone.0042201 PMC3408476

[3] Grant WS (2005) A second look at mitochondrial DNA variability in European anchovy (*Engraulis encrasicolus*): assessing models of population structure and the Black Sea isolation hypothesis. Genetica 25: 293–309. 10.1007/s10709-005-0717-z 16247701

[4] Magoulas A, Castilho R, Caetano S, Marcato S, Patarnello T (2006) Mitochondrial DNA reveals a mosaic pattern of phylogeographical structure in Atlantic and Mediterranean populations of anchovy (*Engraulis encrasicolus*). Mol Phylogenet Evol 39: 734–746. 16515866 10.1016/j.ympev.2006.01.016

[5] Jerôme M, Martinsohn JT, Ortega D, Carreau P, Verrez-Bagnis V, et al (2008) Toward fish and seafood traceability: anchovy species determination in fish products by molecular markers and support through a public domain database. J Agric Food Chem 56: 3460–3469 doi:10.1021/jf703704m 18452298 10.1021/jf703704m

[6] Rea S, Storani G, Mascaro N, Stocchi R, Loschi AR (2009) Species identification in anchovy pastes from the market by PCR-RFLP technique. Food Control 20: 515–520.

[7] Landi M, Garoia F, Piccinetti C, Tinti F (2005) Isolation of polymorphic microsatellite *loci* from the European anchovy, *Engraulis encrasicolus* . Mol Ecol Resour 5: 266–268 doi:10.1111/j.1471–8286.2005.00892.x

[8] Molecular Ecology Resources Primer Development Consortium, Abreu AG, Albaina A, Alpermann TJ, Apkenas VE, et al (2012) Permanent genetic resources added to Molecular Ecology Resources Database 1 October 2011–30 November 2011. Mol Ecol Resources 12: 374–376 doi:10.1111/j.1755–0998.2011.03109.x 10.1111/j.1755-0998.2011.03109.x PMC5479135 22296658

[9] Helyar SJ, Hemmer-Hansen J, Bekkevold D, Taylor MI, Ogden R, et al (2011) Application of SNPs for population genetics of non-model organisms: new opportunities and challenges. Mol Ecol Resour 11: 123–136 doi:10.1111/j.1755–0998.2010.02943.x 21429169 10.1111/j.1755-0998.2010.02943.x

[10] Morin PA, Luikart G, Wayne RK (2004) the SNP workshop group (2004) SNPs in ecology, evolution and Conservation. Trends Ecol Evol 19: 208–216 doi:10.1016/j.tree.2004.01.009

[11] Kumar S, Banks TW, Cloutier S (2012) SNP discovery through Next-Generation Sequencing and its applications. Int J Plant Genomics 2012: 831460 doi:10.1155/2012/831460 23227038 10.1155/2012/831460 PMC3512287

[12] Waples RS, Gaggiotti O (2006) What is a population? An empirical evaluation of some genetic methods for identifying the number of gene pools and their degree of connectivity. Mol Ecol 15: 1419–1439. 16629801 10.1111/j.1365-294X.2006.02890.x

[13] Metzker M (2010) Sequencing technologies – the next generation. Nat Rev Genet 11: 31–46 doi:10.1038/nrg2626 19997069 10.1038/nrg2626

[14] Slate J, Gratten J, Beraldi D, Stapley J, Hale M, et al (2009) Gene mapping in the wild with SNPs: guidelines and future directions. Genetica 136: 97–107 doi:10.1007/s10709–008–9317-z 18780148 10.1007/s10709-008-9317-z

[15] Wang S, Sha Z, Sonstegard TS, Liu H, Xu P, et al (2008) Quality assessment parameters for EST-derived SNPs from catfish. BMC Genomics 9: 450 doi:10.1186/1471–2164–9–450 18826589 10.1186/1471-2164-9-450 PMC2570692

[16] Hale M, McCormick C, Jackson J, DeWoody JA (2009) Next-generation pyrosequencing of gonad transcriptomes in the polyploid lake sturgeon (*Acipenser fulvescens*): the relative merits of normalization and rarefaction in gene discovery. BMC Genomics 10: 203 doi:10.1186/1471–2164–10–203 19402907 10.1186/1471-2164-10-203 PMC2688523

[17] Sánchez CC, Smith TP, Wiedmann RT, Vallejo RL, Salem M, et al (2009) Single nucleotide polymorphism discovery in rainbow trout by deep sequencing of a reduced representation library. BMC Genomics 10: 559 doi:10.1186/1471–2164–10–559 19939274 10.1186/1471-2164-10-559 PMC2790473

[18] Salem M, Rexroad III CE, Wang J, Thorgaard GH, Yao J (2010) Characterization of the rainbow trout transcriptome using Sanger and 454-pyrosequencing approaches. BMC Genomics 11: 564 doi:10.1186/1471–2164–11–564 20942956 10.1186/1471-2164-11-564 PMC3091713

[19] Renaut S, Nolte AW, Bernatchez L (2010) Mining transcriptome sequences towards identifying adaptive single nucleotide polymorphisms in lake whitefish species pairs (*Coregonus spp*. *Salmonidae*). Mol Ecol 19: 115–131 doi:10.1111/j.1365–294X.2009.04477.x 20331775 10.1111/j.1365-294X.2009.04477.x

[20] Hubert S, Higgins B, Borza T, Bowman S (2010) Development of a SNP resource and a genetic linkage map for Atlantic cod (*Gadus morhua*). BMC Genomics 11: 191 doi:10.1186/1471–2164–11–191 20307277 10.1186/1471-2164-11-191 PMC2846918

[21] Seeb JE, Pascal CE, Grau ED, Seeb LW, Templin WD, et al (2011) Transcriptome sequencing and high-resolution melt analysis advance single nucleotide polymorphism discovery in duplicated salmonids. Mol Ecol Resour 11: 335–348 doi:10.1111/j.1755–0998.2010.02936.x 21429141 10.1111/j.1755-0998.2010.02936.x

[22] Everett MV, Grau ED, Seeb JE (2011) Short reads and non-model species: exploring the complexities of next-generation sequence assembly and SNP discovery in the absence of a reference genome. Mol Ecol Resour 11: 93–108 doi:10.1111/j.1755–0998.2010.02969.x 21429166 10.1111/j.1755-0998.2010.02969.x

[23] Milano I, Babbucci M, Panitz F, Ogden R, Nielsen RO, et al (2011) Novel tools for conservation genomics: comparing two high-throughput approaches for SNP discovery in the transcriptome of European hake. PLoS One 6: e28008 doi:10.1371/journal.pone.0028008 22132191 10.1371/journal.pone.0028008 PMC3222667

[24] Vera M, Alvarez-Dios JA, Milian A, Pardo BG, Bouza G, et al (2011) Validation of single nucleotide polymorphism (SNP) markers from an immune Expressed Sequence Tag (EST) turbot, *Scophthalmus maximus*, database. Aquaculture 313: 31–41 doi:10.1016/j.aquaculture.2011.01.038

[25] Helyar SJ, Limborg MT, Bekkevold D, Babucci M, van Houdt J, et al (2012) SNP Discovery Using Next Generation Transcriptomic Sequencing in Atlantic Herring (*Clupea harengus*). PLoS One 7: e42089 doi:10.1371/journal.pone.0042089 22879907 10.1371/journal.pone.0042089 PMC3413699

[26] Roberts SB, Hauser L, Seeb LW, Seeb JE (2012) Development of Genomic Resources for Pacific Herring through Targeted Transcriptome Pyrosequencing. PLoS One 7: e30908 doi:10.1371/journal.pone.0030908 22383979 10.1371/journal.pone.0030908 PMC3288011

[27] Gut IG, Lathrop GM (2004) Duplicating SNPs. Nature Genetics 36 (8): 789–790. 10.1038/ng0804-789 15284844

[28] Zhulidov PA, Bogdanova EA, Shcheglov AS, Vagner LL, Khaspekov GL, et al (2004) Simple cDNA normalization using kamchatka crab duplex-specific nuclease. Nucleic Acids Res 32: e37. 14973331 10.1093/nar/gnh031 PMC373426

[29] Zhu YY, Machleder EM, Chenchik A, Li R, Siebert PD (2001) Reverse transcriptase template switching: a SMART approach for full-length cDNA library construction. Biotechniques 30: 892–897. 11314272 10.2144/01304pf02

[30] Quinlan AR, Stewart DA, Strömberg MP, Marth GT (2008) Pyrobayes: an improved base caller for SNP discovery in pyrosequences. Nat Methods 5: 179–181 doi:10.1038/nmeth.1172 18193056 10.1038/nmeth.1172

[31] Ewing B, Green P (1998) Base-calling of automated sequencer traces using Phred. II. Error probabilities. Genome Res 8: 186–194. 9521922

[32] Blanca JM, Pascual L, Ziarsolo P, Nuez F, Cañizares J (2011) ngs_backbone: a pipeline for read cleaning, mapping and SNP calling using Next Generation Sequence. BMC Genomics: 12, 285. doi: 10.1186/1471–2164–12–285. 10.1186/1471-2164-12-285 PMC3124440 21635747

[33] Lassmann T, Hayashizaki Y, Daub CO (2009) TagDust – A program to eliminate artifacts from next generation sequencing data. *Bioinformatics*, 25, 2839–2840. doi: 10.1093/bioinformatics/btp527. 10.1093/bioinformatics/btp527 PMC2781754 19737799

[34] Milne I, Bayer M, Cardle L, Shaw P, Stephen G, et al (2010) Tablet-next generation sequence assembly visualization. Bioinformatics 26: 401–402 doi:10.1093/bioinformatics/btp666 19965881 10.1093/bioinformatics/btp666 PMC2815658

[35] Langmead B, Trapnell C, Pop M, Salzberg SL (2009) Ultrafast and memory-efficient alignment of short DNA sequences to the human genome. Genome Biol 10: R25 doi:10.1186/gb-2009-10-3-r25 19261174 10.1186/gb-2009-10-3-r25 PMC2690996

[36] Treangen TJ, Salzberg SL (2012) Repetitive DNA and next-generation sequencing: computational challenges and solutions. Nat Rev Genet 13: 36–46 doi:10.1038/nrg3117 10.1038/nrg3117 PMC3324860 22124482

[37] Li H, Handsaker B, Wysoker A, Fennell T, Ruan J, et al (2009) The sequence alignment/map (SAM) format and SAMtools. Bioinformatics 25: 2078–2079 doi:10.1093/bioinformatics/btp352 19505943 10.1093/bioinformatics/btp352 PMC2723002

[38] Conklin D, Montes I, Albaina A, Estonba A (2013) Improved conversion rates for SNP genotyping of non-model organisms. IWBBIO 2013: International Work-Conference on Bioinformatics and Biomedical Engineering, Granada, Spain. ISBN: GR 489/2013, 127–134.

[39] Weir BS, Cockerham CC (1984) Estimating F statistics for the analysis of population structure. Evolution 38: 1358–1370. 28563791 10.1111/j.1558-5646.1984.tb05657.x

[40] Rousset F (2008) genepop'007: a complete re-implementation of the genepop software for Windows and Linux. Mol Ecol Resour 8: 103–106 doi:10.1111/j.1471–8286.2007.01931.x 21585727 10.1111/j.1471-8286.2007.01931.x

[41] Warnes G, Gorjanc G, Leisch F, Man M (2012) genetics: Population Genetics. R package version 1.3.8. Available: http://CRAN.R-project.org/package=genetics. Accessed 2013 May 20.

[42] Piry S, Alapetite A, Cornuet JM, Paetkau D, Baudouin L, et al (2004) GeneClass2: A Software for Genetic Assignment and First-Generation Migrant Detection. J Hered 95: 536–539. 15475402 10.1093/jhered/esh074

[43] Foll M, Gaggiotti OE (2008) A genome scan method to identify selected *loci* appropriate for both dominant and codominant markers: A Bayesian perspective. 10.1534/genetics.108.092221 PMC2567396 18780740

[44] Benjamini Y, Hochberg Y (1995) Controlling the false discovery rate: a practical and powerful approach to multiple testing. J R Stat Soc Ser B 57: 289–300 doi:10.2307/2346101

[45] Meglécz E, Costedoat C, Dubut V, Gilles A, Malausa T, et al (2010) QDD: a user-friendly program to select microsatellite markers and design primers from large sequencing projects. Bioinformatics 26: 403–404 doi:10.1093/bioinformatics/btp670 20007741 10.1093/bioinformatics/btp670

[46] Boeckmann B, Bairoch A, Apweiler R, Blatter MC, Estreicher A, et al (2003) The SWISS-PROT protein knowledgebase and its supplement TrEMBL in 2003. Nucleic Acids Res 31: 365–370 doi:10.1093/nar/gkg095 12520024 10.1093/nar/gkg095 PMC165542

[47] Conesa A, Gotz S, Garcia-Gomez JM, Terol J, Talón M, et al (2005) Blast2GO: a universal tool for annotation, visualization and analysis in functional genomics research. Bioinformatics 21: 3674–3676 doi:10.1093/bioinformatics/bti610 16081474 10.1093/bioinformatics/bti610

[48] Hyman RW, Jiang H, Fukushima M, Davis RW (2010) A direct comparison of the KB™ Basecaller and Phred for identifying the bases from DNA sequencing using chain termination chemistry. BMC Res Notes 3: 257 doi:10.1186/1756–0500–3–257 20932319 10.1186/1756-0500-3-257 PMC3020662

[49] Nielsen R (2004) Population genetic analysis of ascertained SNP data. Hum Genomics 1: 218–224. 15588481 10.1186/1479-7364-1-3-218 PMC3525085

[50] Albrechtsen A, Nielsen FC, Nielsen R (2010) Ascertainment biases in SNP chips affect measures of population divergence. Mol Biol Evol 27: 2534–2547 doi:10.1093/molbev/msq148 20558595 10.1093/molbev/msq148 PMC3107607

[51] Rosenblum EB, Novembre J (2007) Ascertainment bias in spatially structured populations: a case study in the Eastern fence lizard. J Hered 98: 331–336. 17611259 10.1093/jhered/esm031

[52] Nielsen R, Paul JS, Albrechtsen A, Song YS (2011) Genotype and SNP calling from next-generation sequencing data. Nat Rev Genet 12: 443–451 doi:10.1038/nrg2986 21587300 10.1038/nrg2986 PMC3593722

[53] Harismendy O, Ng PC, Strausberg RL, Wang X, Stockwell TB, et al (2009) Evaluation of next generation sequencing platforms for population targeted sequencing studies. Genome Biol 10: R32 doi:10.1186/gb-2009-10-3-r32 19327155 10.1186/gb-2009-10-3-r32 PMC2691003

[54] Pérez-Enciso M, Ferreti L (2010) Massive parallel sequencing in animal genetics: wherefroms and wheretos. Animal Genet 41: 561–569 doi:10.1111/j.1365–2052.2010.02057.x 20477787 10.1111/j.1365-2052.2010.02057.x

[55] Martin JA, Wang Z (2011) Next-generation transcriptome assembly. Nat Rev Genet 12: 671–682 doi:10.1038/nrg3068 21897427 10.1038/nrg3068

[56] Alkan C, Sajjadian S, Eichler EE (2011) Limitations of next-generation genome sequence assembly. Nat Methods 8: 61–65 doi:10.1038/nmeth.1527 21102452 10.1038/nmeth.1527 PMC3115693

[57] Gut IG, Lathrop GM (2004) Duplicating SNPs. Nat Genet 36: 789–790. 15284844 10.1038/ng0804-789

[58] Bainbridge MN, Wang M, Wu Y, Newsham I, Muzny DM, et al (2011) Targeted enrichment beyond the consensus coding DNA sequence exome reveals exons with higher variant densities. Genome Biol 12(7): R68 doi:10.1186/gb-2011-12-7-r68 21787409 10.1186/gb-2011-12-7-r68 PMC3218830

[59] Moen T, Delghandi M, Wesmajervi MS, Westgaard JI, Fjalestad KT (2009) A SNP/microsatellite genetic linkage map of the Atlantic cod (*Gadus morhua*). Animal Genet 40: 993–996 doi:10.1111/j.1365–2052.2009.01938.x 19694651 10.1111/j.1365-2052.2009.01938.x

[60] Kraus RHS, Kerstens HHD, Van Hooft P, Crooijmans RP, Van Der Poel JJ, et al (2011) Genome wide SNP discovery, analysis and evaluation in mallard (*Anas platyrhynchos*). BMC Genomics 12: 150 doi:10.1186/1471–2164–12–150 21410945 10.1186/1471-2164-12-150 PMC3065436

[61] Kerstens HHD, Crooijmans RPMA, Veenendaal A, Dibbits BW, Chin-A-Woeng TF, et al (2009) Large scale single nucleotide polymorphism discovery in unsequenced genomes using second generation high throughput sequencing technology: applied to turkey. BMC Genomics 10: 479 doi:10.1186/1471–2164–10–479 19835600 10.1186/1471-2164-10-479 PMC2772860

[62] Li X, Acharya A, Farmer AD, Bharti AK, Kramer RS, et al (2012) Prevalence of single nucleotide polymorphism among 27 diverse alfalfa genotypes as assessed by transcriptome sequencing. BMC Genomics 13: 568 doi:10.1186/1471–2164–13–568 23107476 10.1186/1471-2164-13-568 PMC3533575

[63] Studer B, Byrne S, Nielsen RO, Panitz F, Bendixen C, et al (2012) A transcriptome map of perennial ryegrass (*Lolium perenne* L.). BMC Genomics 13: 140 doi:10.1186/1471–2164–13–140 22513206 10.1186/1471-2164-13-140 PMC3483695

