# Supplementary figures and images for: SNP Discovery in European Anchovy (Engraulis encrasicolus, L) by High-Throughput Transcriptome and Genome Sequencing
Source: PLoS One. 2013 Aug 1;8(8):e70051. doi: 10.1371/journal.pone.0070051 (PMC3731364; doi:10.1371/journal.pone.0070051)

Before trimming

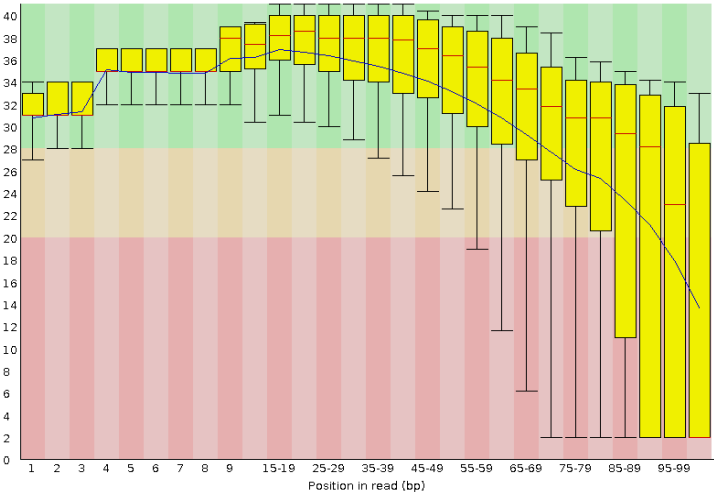

After trimming

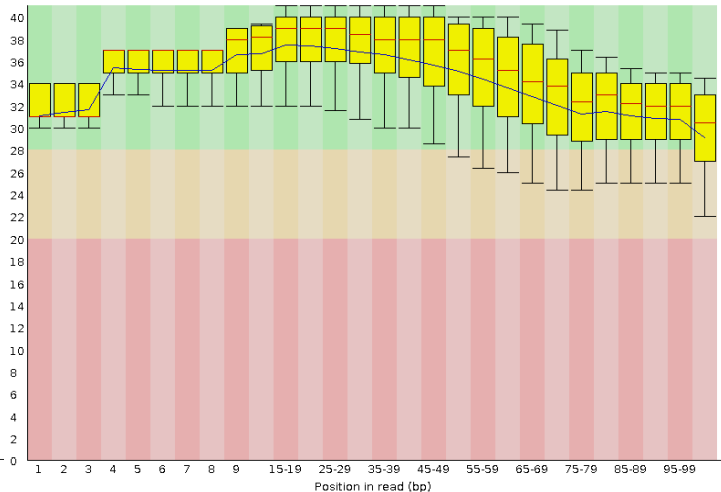

Supplement: Figure S1 — FastQC tool generated quality plot for CAD-1 individual genome sequences before (left) and after (right) contaminants removal, and length and quality trimming. In the plot X axis represents position in the read (bp) from 0 to 100, and Y axis represents quality values in Phred+33 scale (from 0 to 40). (PDF) [file pone.0070051.s001.pdf]

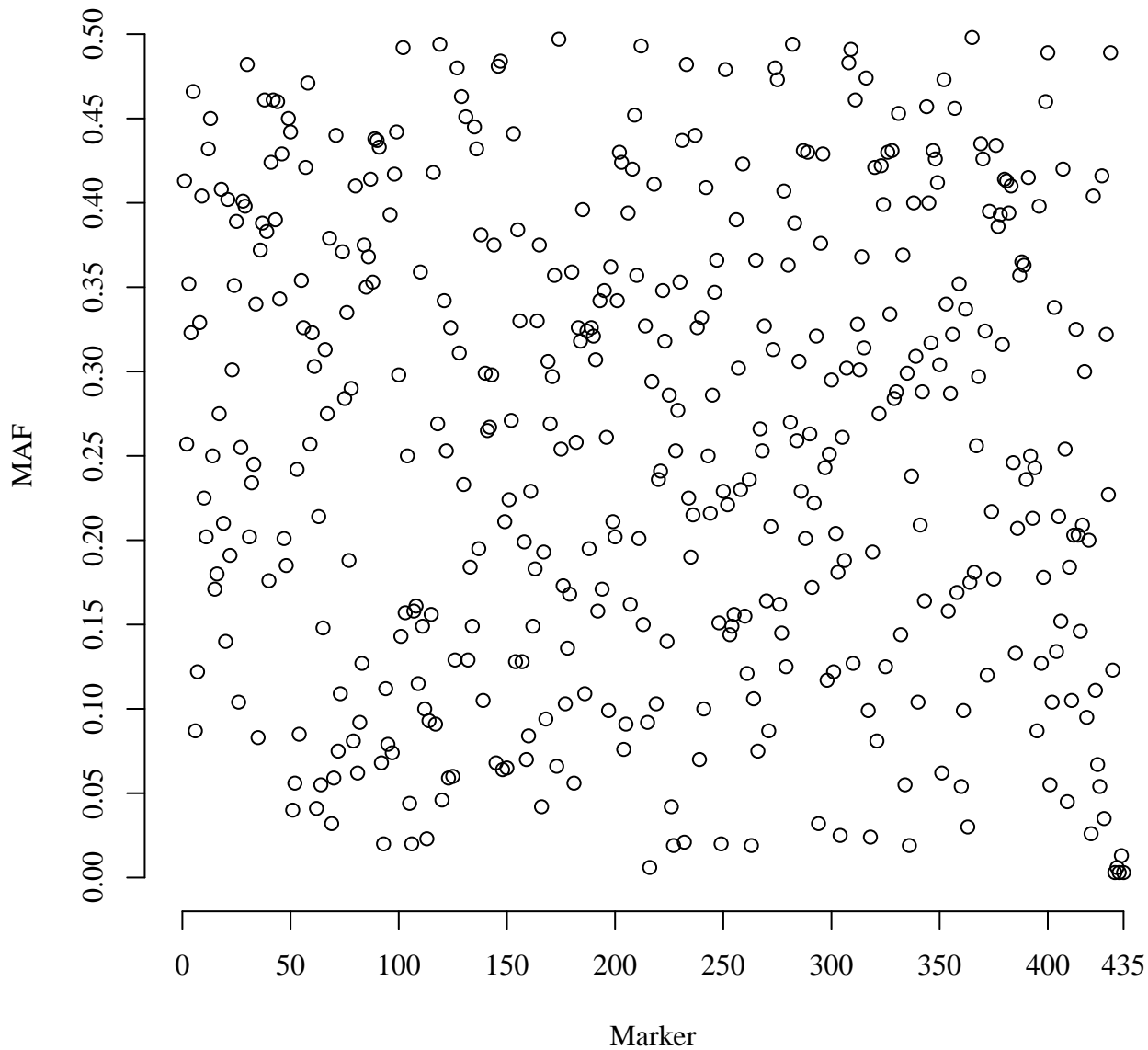

Supplement: Figure S2 — Minor allele frequencies (MAF) values obtained from 435 independent validated markers (in H-W equilibrium or not). (PDF) [file pone.0070051.s002.pdf]

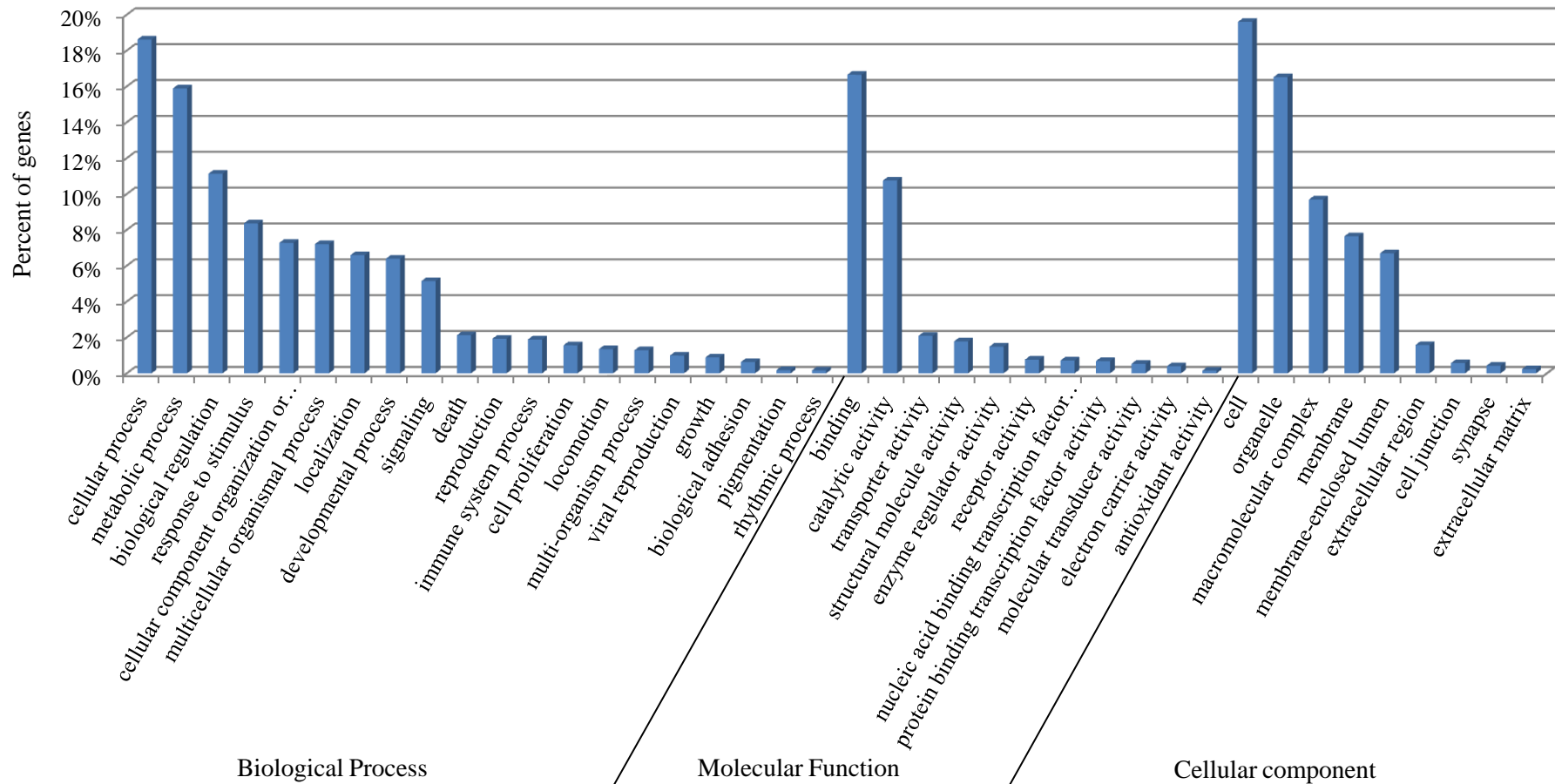

Supplement: Figure S3 — Level 2 gene ontology terms, divided in the three categories, and the percentage of Engraulis encrasicolus genes for each term. (PDF) [file pone.0070051.s003.pdf]
